# Technical aspects of neuraxial analgesia during labor and maternity care: an updated overview

**DOI:** 10.1186/s44158-025-00224-3

**Published:** 2025-01-29

**Authors:** Antonio Coviello, Carmine Iacovazzo, Maria Grazia Frigo, Marilena Ianniello, Dario Cirillo, Giuseppe Tierno, Andrea Uriel de Siena, Pasquale Buonanno, Giuseppe Servillo

**Affiliations:** 1 Department of Neurosciences, Reproductive and Odontostomatological Sciences, University of Naples “Federico II”, via Sergio Pansini 5, Naples, 80100 Italy; 2 UOSD, Obstetric Anesthesia and Resuscitation, Isola Tiberina Hospital - Gemelli Isola, Rome, 00186 Italy

**Keywords:** Epidural analgesia, Labor analgesia, Maternal–fetal safety, Labor pain

## Abstract

Labor analgesia is increasingly widespread throughout the world with a rate ranging from 10 to 60%. The benefits regarding clinical and non-clinical maternal–fetal outcomes are currently discussed in international scientific literature. Even stage of labor needs a different and appropriate approach to control the pain; however, different techniques are reported in literature. The following study intends to give a brief overview of the characteristics of the different neuraxial and non-neuraxial techniques currently available and the non-technical skills necessary for effective assistance to pregnant women, providing insights on the topic to understand critical issues at the same time. After bibliographic research since 2018 to 2023, many randomized controlled trials, literature reviews, systematic reviews, and metanalysis were evaluated to create this brief overview. The following pharmacological and non-pharmacological approaches were assessed: spinal techniques, such as epidural analgesia (EA), combined spinal-epidural (CSE), dural puncture epidural (DPE), and continuous spinal anesthesia (CSA); pharmacological administration of nitrous oxide (N_2_O) and systemic opioids (morphine, fentanyl, and pethidine); as the third one transcutaneous electric nerve stimulation (TENS), acupressure/acupuncture, aromatherapy, and breathing exercises. All the assessed approaches are relatively safe and effective, but the association of technical and non-technical skills is needed to improve the maternal and fetus outcome. More studies are needed to clarify what is the best approach to labor analgesia.

## Study design

This review was aimed to provide a brief overview of neuraxial techniques with a look at the non-neuraxial one and non-technical skills and the respectful maternity care recommended by the WHO to guarantee women dignity, privacy, and continuous support during labor and childbirth. To gain a better understanding of the critical issues in current literature, insights on the topic were provided.

## Introduction

Nowadays, labor analgesia is a growing practice and neuraxial analgesia (NA) is recognized as the most effective technique known. The World Health Organization (WHO) recommends epidural analgesia (EA) as the gold standard for labor analgesia (estimated range of use 10–64% in high-income countries) [1, 2]. It consists of lowering the pain associated with childbirth labor by improving maternal–fetal clinical outcomes as well as the maternal pain experience. Each stage of labor involves different nerve fibers and roots that are the target of labor analgesia. It could be performed (neuraxial and non-neuraxial) with pharmacological administration and non-pharmacological techniques; the neuraxial approaches include EA, combined spinal-epidural (CSE), dural puncture epidural (DPE), and continuous spinal anesthesia (CSA); the use of inhaled nitrous oxide (N_2_O) and systemic opioids (morphine, meperidine, fentanyl, and remifentanil); instead, the non-pharmacological approaches include transcutaneous electric nerve stimulation (TENS), acupressure/acupuncture, aromatherapy, and breathing exercises [3]. Many brief reviews produced on labor pain relief have focused on the description of neuraxial techniques (Halliday et al., Kearns and Lucas, Toledano and Leffert, and Chau and Tsen) with interesting comparisons of their benefits and side effects both on mother and fetus [4–7]; these reviews highlighted the importance of this argument but they did not consider the non-pharmacological approaches and the importance of the maternal choice. With this minireview, the authors will provide a brief overview of NA techniques with a look at the non-neuraxial one and non-technical skills (organizational, communication, emotional, and structural) and the respectful maternity care (RMC) recommended by the WHO to guarantee women dignity, privacy, and continuous support during labor and childbirth [1, 8].

## Methods

A bibliographic search was conducted from May to October 2023 using Medline/PubMed, Embase, and the Cochrane Library. The following search terms (MeSH) were used: "pregnancy", "labor analgesia", "epidural analgesia", "combined spinal-epidural analgesia", "labor pain", "maternal safety", "fetal safety", "labor analgesia outcomes", and "COVID-19 pregnant". The included studies were randomized controlled trials (RCTs), reviews of literature (systematic or not), and meta-analyses published between 2018 and 2023, written in English or Italian, and available in full text.

### Labor

Labor pain is very intense and correlates with psychological and physiological mechanisms. Three stages can be distinguished during labor: the first stage from the onset of cervical dilation to full dilation, the second stage from full dilation to the expulsion of the fetus, and the third stage from delivery to the expulsion of the placenta. The first stage or dilatative phase is itself divided into two phases: (1) latent phase, characterized by nonrhythmic contractions, not always perceived as painful, and variable changes in the cervix, including some degree of applanation and slow progression of dilatation; (2) active phase, characterized by rhythmic active contractions (at least 3 regular contractions every 10 min, increasing intensity, and reducing intervals between contractions), which are painful and result in changes in the cervix including substantial applanation and progressive dilatation. The dilatative phase ends when the cervix reaches a dilation of 10 cm and the second stage of labor (or expulsive phase) begins. Between the end of the dilatative phase and the beginning of the expulsive phase, there is another phase called “transition”, where the fetal head will have to adapt to the maternal tissues as it progresses through the birth canal.

Labor pain is composed of a visceral component in the first stage and a predominant somatic component in the second stage. The visceral component is caused by cervical stretching, and C-fibers transmit it via T10–L1 roots, referred to T10–T12 dermatomes with pain perceived as back pain and lower abdominal pain. The somatic component is localized to the vagina, rectum, and perineum, A-delta fibers transmit it via S2–S4 and L1–L2 roots; the somatic pain is transmitted via the spinothalamic tract to the hypothalamus and limbic system; this integration with limbic system explains the emotional implication of labor pain. The different pain characteristics explain the reason for the different response to drugs depending on the different pathways involved: visceral pain responds to opioids while somatic pain responds to local anesthetics (LAs) [8]. The neuraxial approach for labor analgesia is based on this analysis.

### Neuraxial procedure in labor

The perfect technique for analgesia during labor should ensure good and rapid efficacy, no side effects, simple to perform, and predictable effects on mother and fetus. NA represents the most widely used and effective approach to pain management during labor. The main techniques used include EA, CSE, DPE, and CSA [7] (see Table 1).

**Table 1:** Neuraxial techniques, advantages/disadvantages, and side effects related (data drawn from the current literature)

	**Advantages**	**Disadvantages**	**Obstetric side effects**	**Maternal side effects**	**Fetal side effects**
**EA**	Pain relief, less anxiety, reduction of catecholaminergic burst (tocolytic action and risk factor for postpartum depression), possible conversion to epidural anesthesia in emergency.	Slower analgesia onset, high anesthetic consumption, high hospital costs, higher incidence of motor block, higher catheter failure and replacement rates, asymmetric block.	Second labor stage prolongation.	Self-limiting hypotension.	Self-limiting bradycardia.
**CSE**	Faster analgesia onset, greater maternal satisfaction rate, lower catheter failure and replacement rates, possible conversion to epidural anesthesia in emergency.	At higher anesthetic concentrations: higher motor block rate. Itching (with intrathecal opioids)	Second labor stage prolongation.	At higher anesthetic concentrations: increased maternal hypotension, headache, nausea and vomiting.	At higher anesthetic concentrations: transient fetal bradycardia.
**DPE**	Faster analgesia onset, maternal satisfaction, lower catheter failure, possible conversion to epidural anesthesia in emergency.	PDPH risk.	Second stage prolongation.	Less cardiovascular side effects incidence.	Less cardiovascular side effects incidence.
**CSA**	Rapid onset, symmetrical sensory blockade, low anesthetic dosages and possible conversion to spinal anesthesia in emergency.	PDPH, cauda equina and transient neurological syndrome risk.	Lower prolongation of second stage.	Higher maternal satisfaction in the first 24 h postpartum, hemodynamic stabilization in hypertensive patients.	

### General implications of labor analgesia

Previous studies have focused on the Robson 1 class of pregnant women (nulliparous, single cephalic pregnancy, at least 37 weeks gestation, spontaneous labor). It appears that the acceleration of complete dilation does not coincide with an accelerated fetal descent or involvement of the birth canal [9]. Eguchi et al. reported an increased likelihood of instrumental vaginal deliveries among women who received labor analgesia; however, there was no significant increase in the rates of cesarean sections overall [10]. This finding is consistent with the review by Landau et al., which concluded that while labor analgesia may prolong the second stage of labor, it does not correlate with a higher risk of cesarean delivery [11].

Concerns regarding the potential impact of labor analgesia on neonatal well-being have been addressed in several studies. Eguchi et al. found no significant differences in Apgar scores between neonates born to mothers who received labor analgesia and those who did not, suggesting that labor analgesia does not adversely affect immediate neonatal health [10].

### EA

The most widely used and effective technique from 1960 to the present is EA for labor pain relief. It is a central nerve block technique considered the gold standard for labor analgesia and recommended by the WHO [1], although the frequency of its use still widely varies due to concerns about unintended adverse effects [12]. However, many meta-analyses and RCTs have been conducted confirming the safety of central nerve block technique about the number of cesarean sections, neonatal outcomes, and assisted vaginal deliveries as the 2018 Cochrane review by Anim-Somuah et al. (40 RCT; > 11,000 parturient); two of the most discussed disadvantages of EA are the prolongation of the second stage of delivery and the delayed, inadequate or absent sacral block [12]. About the first topic, a systematic review by Ashagrie et al. stated that the prolongation of the second stage of labor is related to multiple causes and that no statistically significant association occurs between NA and this condition [13]. EA was also compared with DPE technique by Lin et al. [14]. The comparison was based on the hypothesis that the DPE assists the diffusion from epidural space to subarachnoid one; indeed, the DPE practiced with 25-Gauge Whitacre needle seemed to guarantee a better blockade of the sacral fibers with comparable incidence of side effects [14]. While for the second topic, a RCT by Malik et al. showed that the incidence of S2 blockade at 30 min after 10 mL of 0.125% bupivacaine was similar whether the epidural catheter was inserted at the L2-L3 or L3-L4 or at L5-S1 (81% vs. 91%, *p*-value = 0.24) during labor analgesia [15]. Regarding this topic, Lin et al. showed that, comparing DPE and EA at 20 and 30 min after LA bolus administration, S2 blockade was more frequently observed in DPE than EA (*P* = 0.006) while no difference was noted in blockage asymmetry at 10 min (RR = 0.530; CI 0.226–1.241; *P* = 0.134) [14].

### CSE

CSE is usually performed with a needle-through-needle technique that allows the administration of analgesic drugs both in the spinal and epidural space. CSE has gained popularity because of its advantages over traditional EA at high concentration of LAs, such as a faster effective analgesia, a safe ambulation during labor, as well as the availability of an epidural catheter for the maintenance of analgesia target on the type of pain of each stage or management of unplanned cesarean deliveries. Indeed, no differences were found by Aragão et al. between EA with low concentration of LAs (0.125–0.0625% bupivacaine and 0.17% ropivacaine) and CSE about the incidence of cesarean section, forceps’ use, oxytocin doses, side effects (hypotension, urinary retention, nausea and vomiting, headache post neural puncture), and neonatal clinical conditions (umbilical cord pH, Apgar score, NICU admission) [16]. However, a meta-analysis by Grangier et al. showed an increased risk of nausea/vomiting (RR 1.31, CI 1.0 to 1.72), itching (RR 4.26, CI 2.59 to 7.0), fetal bradycardia (RR 2.38, CI 1.57 to 3.62), and a higher hypotension rate after CSE opioid administration (RR 1.54, 1.22 to 1.93; *p*-value = 0.02 for subgroup difference) in patients who received CSE instead of EA [17]. However, Hembrador et al. found no differences between increasing CSE fentanyl doses of 2.5 mcg, 5 mcg, 10 mcg and 15 mcg in prolonged fetal decelerations rates (respectively 4.4%, 2.3%, 7.6%, 3.0%, *p*-value = 0.11), emergency cesarean delivery, extent of pain reduction, itching, or maternal hypotension episode [18].

Despite the increasing use of this technique and numerous published research papers, the optimal intrathecal drug regimen has not yet been determined. The choice between conventional EA and CSE is often conditioned by the clinical situation, institutional protocols, equipment available, and operator preference/experience. A recent expert review focused on EA and CSE analgesia during labor reported that CSE is possible related with fetal bradycardia events, even if the precedent literature did not confirm it [19, 20]. According to our experience, the use of CSE or DPE is safe and effective both for the mother and fetus; both procedures allow the reduction of the LA concentrations. Considering our expertise, CSE is also associated with faster pain relief due to the use of intrathecal sufentanyl in association or not with low dose of ropivacaine; it is important to underline that fast pain relief could reduce the adrenergic tone in favor of noradrenergic one: this condition could induce uterine hypertonia and fetal bradycardia, consequently. The possibility of hypertonia is higher as higher mother’s pain during the first stage of labor due to the pain stress-induced catecholamine release; if the pain is too high, DPE guarantees a slow pain relief that allows a re-balance of catecholamines’ level.

A recent meta-analysis of RCTs by Zhi et al. shows that sufentanyl compared with fentanyl provides longer duration of spinal analgesia (95% CI 21.82 to 28.98 min; *p* < 0.001), lower incidence of nausea and vomiting, and a better Apgar score at 5 min (WMD 0.10 [95% CI 0.05 to 0.16]; *p* = 0.0002), while the rate of respiratory depression was nonsignificant in both groups (RR 0.80 [95% CI 0.23 to 2.84]; *p* = 0.73) [21]. Despite proven efficacy and increasing research on the topic, the optimal intrathecal drug regimen for CSE has not yet been defined. For this reason, institutional protocols differ with regard to the preference of technique to be used based on the clinical situation, available devices, and operators’ experience.

#### DPE

DPE technique was developed by Cappiello and colleagues and allows to enhance the traditional EA by passing the anesthetic mixture into the subarachnoid space thorough an orifice performed in the dura mater [22]. It provides an improved central nerve block with a lower incidence of side effects associated to EA (slow onset, unilateral, and sacral block sparing) and to CSE (fetal bradycardia and itching), although, again, the scientific literature does not agree on the actual safety of this technique. Indeed, a systematic review by Yin et al. found no differences between DPE and EA technique either about the incidence of spontaneous vaginal birth (RR 1.01; *p*-value = 0.71) or of cesarean birth (RR 0.89; *p*-value = 0.41), neither about the unilateral block recurrence (RR 0.60; *p*-value = 0.10; *I*^2^ = 68%) nor of motor block (RR 0.74; *p*-value = 0.35; *I*^2^ = 0%) [23]. However, DPE resulted in a lower incidence of nausea and vomiting than EA (RR 0.35; 95% CI 0.14, 0.91; *p*-value = 0.03; *I*^2^ = 0%) [23].

#### CSA

CSA, conducted by administrating analgesics through an intrathecal catheter, could be a new and valuable method for labor analgesia as stated by Ji et al. [24]. Originating as a rescue technique after inadvertent dural puncture (IDP), it is currently considered a promising technique because of its advantages, such as lower drug usage, more precise analgesic effect and circulatory system stabilization as shown by Han and Xu [25]. Although the risk of post-dural puncture headache (PDPH) and neurological complications compromises the wide use of this technique, the advantages listed are to be considered in case-by-case evaluation.

### Timing of labor analgesia

The timing of labor analgesia is as important as its technical aspects and has been a significant topic of discussion in recent years, shaped by emerging evidence. There are two main approaches: early initiation of analgesia (when cervical dilation is less than 4 cm) and late initiation (when cervical dilation is 4 cm or more). A Cochrane meta-analysis indicates that there is no significant difference between these two approaches in terms of cesarean section rates or maternal and fetal outcomes [20, 26]. However, some evidence suggests that initiating analgesia earlier may prolong certain phases of labor, with 6 cm of cervical dilation proposed as an optimal threshold for starting analgesia [27]. Despite these findings, the American College of Obstetricians and Gynecologists (ACOG) recommends that a maternal request alone is a valid reason to begin analgesia, provided there are no medical contraindications [28]. This patient-centered approach has gained increasing prevalence in recent years.

### Neuraxial block‐related complications

Despite the strength of the scientific literature on the subject, there is still a great deal of fear and mistrust about NA techniques due to the possible complications among both pregnant women and healthcare team. One of the major concerns relates to the risk of epidural hematoma. Currently, the incidence of this complication is 1 in 170,000 with variations related to the center where NA is performed [4]. Indeed, altered coagulation status, pre-existing coagulopathies or complications for example HELLP syndrome (hemolysis, elevated liver enzymes, and low platelets) are common in pregnant women. For these reasons, the Consensus Statement led by the Society for Obstetric Anesthesia and Perinatology, in collaboration with the American Society of Regional Anesthesia and Pain Medicine, the ACOG, and the Society for Maternal–Fetal Medicine has set the platelet count cut-off for performing epidural approach at 70–75 × 10^9^/L [29]. Another noteworthy complication is PDPH. The incidence of PDPH is reported from < 2% up to 40%, depending on the expertise of the center and the number of labor analgesia performed per year, similar among CSE, DPE and EA [30]. In a large meta-analysis of 57 studies, Heesen et al. found a statistically significant reduced incidence of PDPH using pencil point needles than cutting spinal ones (RR 0.41; 95% CI, *p*-value < 0.001) without significant differences between different needle calibers (range 22–27 Gauge) [31]. Otherwise, significantly higher rates of PDPH occurred with CSA procedure with a small-diameter catheter standing in situ less than 12 h [32]. Indeed, in case of IDP, the approaches described are epidural catheter re-site in a different intervertebral space with a 10% risk of new IDP or catheter placement in the subarachnoid space through the dural hole leading then a CSA. There is no consensus on the most effective practice, so the decision is based on institutional protocols. The main risks remain complete spinal block with severe hypotension. Nevertheless, CSA could be the most effective option in case of multiple prolonged catheter positioning attempts [33].

Maternal hypotension is a well-documented side effect of neuraxial labor analgesia. It occurs due to the blockade of the sympathetic nervous system, resulting in arterial and venous dilation and a subsequent reduction in venous return, leading to “functional” hypovolemia [34]. While lumbar epidurals theoretically carry a lower risk of sympathectomy compared to thoracic epidurals, studies show that maternal hypotension remains a significant concern [20]. For example, a randomized controlled trial (RCT) by Gambling et al. reported that 14% of patients receiving CSE (10 μg of sufentanil followed by epidural maintenance with bupivacaine and fentanyl) required vasopressor therapy for hypotension, whereas no such cases were observed in the comparator group using intravenous meperidine [35]. Hypotension is generally transient and can be effectively managed with fluid preloading or co-loading, the administration of vasopressors, and patient positioning adjustments. Importantly, in most cases, maternal hypotension does not lead to adverse fetal outcomes when appropriately treated [20].

Epidural-related maternal fever (ERMF) is another side effect associated with labor epidurals, stemming from a poorly understood inflammatory process [32]. It is not infectious in nature, as evidenced by the ineffectiveness of broad-spectrum antibiotics in preventing or treating it. Studies have reported an incidence of ERMF ranging from 15 to 25%, depending on the population and definitions used for fever [32, 36]. Glucocorticoid therapy has shown promise in reducing the incidence of ERMF, suggesting an inflammatory etiology [20]. ERMF has been associated with neonatal complications such as depressed Apgar scores, suspected sepsis, and increased NICU admissions, though causation is not definitively established. Notably, fever typically resolves quickly after delivery, with limited long-term effects observed in neonates [20].

Fetal adverse effects linked to labor analgesia are generally mild and transient. These include heart rate abnormalities such as bradycardia, decelerations, and reduced variability, which may result from maternal hypotension or sudden pain relief that alters circulating catecholamine levels [20, 37]. These effects are typically self-limiting and can be managed through interventions like fluid boluses, maternal repositioning, or the administration of tocolytics to reduce uterine contractions and restore placental perfusion. Additionally, NA is associated with improved fetal acid–base balance compared to systemic opioid analgesia. Studies show that labor epidurals lead to less fetal acidosis, likely due to better intervillous blood flow and reduced fetal stress [20, 38].

Overall, while labor analgesia is associated with potential side effects, these are generally manageable with appropriate interventions. The benefits of effective pain control and improved maternal and fetal outcomes outweigh these risks when analgesia is administered under careful supervision and with adequate preparation for managing complications.

### Drug choices: LAs and adjuvants

The primary goal in selecting drugs for EA during labor is to achieve effective pain relief while minimizing motor blockade and adverse effects. This is typically accomplished by combining low concentrations of LAs with opioids. Regarding LAs, the most used are ropivacaine and levobupivacaine used at low concentrations (e.g., 0.0625 to 0.125%) to provide sensory blockade with minimal motor impairment. This property to provide a sensory block, especially of pain fibers, without motor block is called “sensory-motor block dissociation”: this effect is particularly important to preserve Ferguson’s reflex, implicated in delivery progression [39, 40]. Ropivacaine is often preferred due to its reduced cardiotoxicity and lower propensity for motor block compared to bupivacaine.

The addition of adjuvants to the anesthetic mixture used in NA is known to significantly lower the rate of adverse effects and complications due to high dosages of LAs such as LAST (local anesthetics systemic toxicity), motor block, and cesarean sections. Several adjuvants such as opioids, alpha agonists, neostigmine and epinephrine have gained popularity and are routinely used. Sufentanyl and fentanyl are the most effective lipophilic opioids used in labor epidural and spinal analgesic techniques [41]. As demonstrated by Väänänen et al. in their RCT, no differences were reported about analgesic power between spinal or epidural sufentanyl and fentanyl, but a slower onset and lower incidence of itching was observed in epidural opioid groups compared to the spinal ones [41]. Additionally, none of these synthetic opioids caused pathological changes in cardiotocography within 30 min after administration [21]. Nonetheless, a prolonged analgesia and better Apgar score at 5 min was observed in sufentanyl group as shown by a metanalysis by Zhi et al. [21]. Morphine, on the other hand, is less widely used during labor because of a higher risk of maternal and neonatal side effects at the effective analgesic dose [42]. Another reason has to do with the fact that morphine and meperidine are also used for systemic analgesia, often leading to neonatal depression because of the high rate of drug crossing the placental barrier [42]. A recent meta-analysis of Cochrane by Smith et al. analyzed the use of intramuscular (IM) opioids (pethidine, tramadol, morphine, and others) and intravenous (IV) opioids (fentanyl, pethidine, morphine, and others) in treating labor analgesia [43]. The main findings were that opioids were related with better control pain and maternal satisfaction in comparison with placebo; IM or IV pethidine seemed to be more effective than the other opioids if administered in the same route in control labor pain [43]. All the findings were with low or very low grade; moreover, the maternal side effects like nausea and vomit were more present in the opioid group and the babies’ outcome were not well assessed [43].

The use of alpha-agonists is increasing. On the one hand, an RCT by Lee et al. on the administration of clonidine with LAs has found differences neither about analgesic effectiveness nor about sedation at 15 min between clonidine and fentanyl mixed with LAs (66.0% after clonidine vs 74.5% after fentanyl, *p*-value = 0.48) [44], although an increased rate of maternal hypotension with epidural clonidine has banned this drug from use in labor NA [45]. On the other hand, dexmedetomidine added to LAs has same analgesic effects and rate of adverse effects (itching and motor blockade) of opioids with LA or LA alone [45, 46], although the analysis led by Li et al. raises some concerns regarding bradycardia and maternal hypotension [45]. Adrenergic alpha-2 agonists have a direct effect on the human myometrium, which has important clinical implications for obstetric analgesia. Both dexmedetomidine and clonidine have been shown to increase uterine contractility. This finding is particularly significant in the context of obstetric anesthesia. In cases where labor analgesia is administered early in labor, when uterine contractions are still weak, alpha-2 agonists may be recommended as adjuncts in NA [47]. There are currently not enough studies to recommend its use. However, as regards neostigmine, interesting evidence has been acquired. A RCT by Abeer et al. concluded that the addition of neostigmine significantly reduced the total dose of LA, resulted in a more rapid onset of sensory blockade, prolonged duration of analgesia, and a lower labor duration. No differences were found regarding the incidence of motor blockade, hemodynamic instability, and Apgar scores at 1 and 5 min or maternal–fetal outcomes [48]. Instead of a meta-analysis by Tschopp et al. shows the analgesic effectiveness of epidural epinephrine; on the other hand, it is associated with a prolonged motor blockade, so its use is not recommended [49].

### Maintenance techniques

Maintenance techniques for NA during labor play a critical role in balancing effective pain relief with minimizing side effects. Continuous epidural infusion (CEI), which delivers a constant infusion of analgesic solution, is straightforward and provides steady analgesia but can result in higher drug consumption and an increased risk of motor block [50, 51]. Patient-intermittent epidural bolus (PIEB) is a more advanced technique that administers scheduled boluses of analgesic solution, promoting a more uniform spread within the epidural space [52–54]. When compared to CEI, PIEB has been shown to enhance analgesic efficacy, reduce LA usage, and decrease motor blockade [51, 55]. Combining PIEB and patient-controlled epidural analgesia (PCEA), which allows patients to self-administer boluses within prescribed limits, further improves analgesic outcomes and maternal satisfaction, while reducing the need for clinician interventions, made it useful for setting with low anesthesiologist resource [54, 56]. In low-resource setting, advanced infusion pumps and monitoring equipment may be limited, CEI remains a feasible option, though careful management is essential to optimize analgesia and minimize complications.

### Other pharmacological and non-neuraxial labor pain relief methods

There are a variety of options for the pain relief of childbirth that can be freely chosen by the mother. Some methods are non-pharmacological and include relaxation techniques (yoga, music therapy, hypnosis), manual techniques (massage, cold or hot compresses, acupuncture), TENS and aromatherapy used effectively both complementary and alternatively to pharmacological techniques [33].

Non-neuraxial methods studied in the literature include the administration of opioids, acetaminophen and nonsteroidal anti-inflammatory drugs (NSAIDs) systemically and inhaled N_2_O [33].

The recent reviews show that the use of opioids (morphine, meperidine, fentanyl, and remifentanil) as well as acetaminophen and NSAIDs raise concerns about maternal safety (excessive sedation, nausea, vomiting, itching) and neonatal adverse effects (hypoxia, poor Apgar score at birth) [33].

Thanks to technological advances, recently virtual reality (VR) became a considered strategy for labor analgesia. VR is gaining recognition as a promising non-pharmacological approach for managing labor pain, utilizing immersive distraction techniques to alter pain perception by engaging the brain in a simulated environment. Musters et al. demonstrated that both guided meditation and interactive VR experiences significantly decreased Numeric Rating Scale (NRS) pain scores by 26 and 19%, respectively, with participants favoring guided meditation due to its calming nature and minimal physical effort [57]. Similarly, Baradwan et al. conducted a meta-analysis, finding that VR significantly reduced pain scores (mean difference =  − 1.40, *p* < 0.001), decreased anxiety levels (SMD =  − 1.15, *p* = 0.03), and markedly improved satisfaction with the childbirth experience (mean difference = 15.58, *p* = 0.004) [58]. In addition to its analgesic benefits, VR provides advantages over pharmacological methods, such as the absence of risks like sedation, maternal hypotension, or neonatal respiratory depression, enhancing maternal satisfaction. These findings underscore VR’s promise as an innovative, non-invasive method to improve labor experiences for women worldwide.

The integration of artificial intelligence (AI) into labor analgesia holds significant promise for enhancing maternal care by enabling personalized and adaptive pain management strategies [59]. AI-powered tools can analyze vast amounts of clinical data, including patient-specific factors such as pain tolerance, medical history, and labor progression, to recommend optimal analgesic techniques and dosages. Moreover, AI has the potential to monitor real-time physiological parameters, predict pain intensity, and adjust medication delivery through advanced systems like PCEA [60]. These innovations aim to improve the safety, efficiency, and satisfaction of labor analgesia, reducing the likelihood of adverse effects and overmedication.

### Other than technical skills

WHO, alongside technical skills and the appropriate choice of analgesic practice for labor, underlines the importance of a competent and motivated staff, a per-established referral plan and an emotional support [1]. Among the most relevant recommendations, the need for the woman’s free choice, informed consent with an effective communication “simple and culturally acceptable”, the need to guarantee more choices for the management of labor pain and respect for the refusal to analgesia itself are identified [1].

In addition, what the WHO emphasizes is the establishment of a suitable environment for a positive childbirth experience: a personal delivery room, a “companion of choice” (partner, friend, relative or a doula) for the entire duration of labor and a dedicated midwife who guides and assists her professionally [1].

The WHO stresses also the importance for institutions to consider and acquire high-quality intra-partum care models by focusing on community sensitization about the RMC, facility-based practices (good communication, choice of best position and labor pain relief technique) and unnecessary birth practices such as freely use of episiotomy, fundal pressure and routine amniotomy [1].

An effective way to implement respect for maternity could be information about the rights of the mother and the unborn child through online outreach and through institutional meetings by health professionals and mothers sharing their birth experiences.

**Fig. 1:**
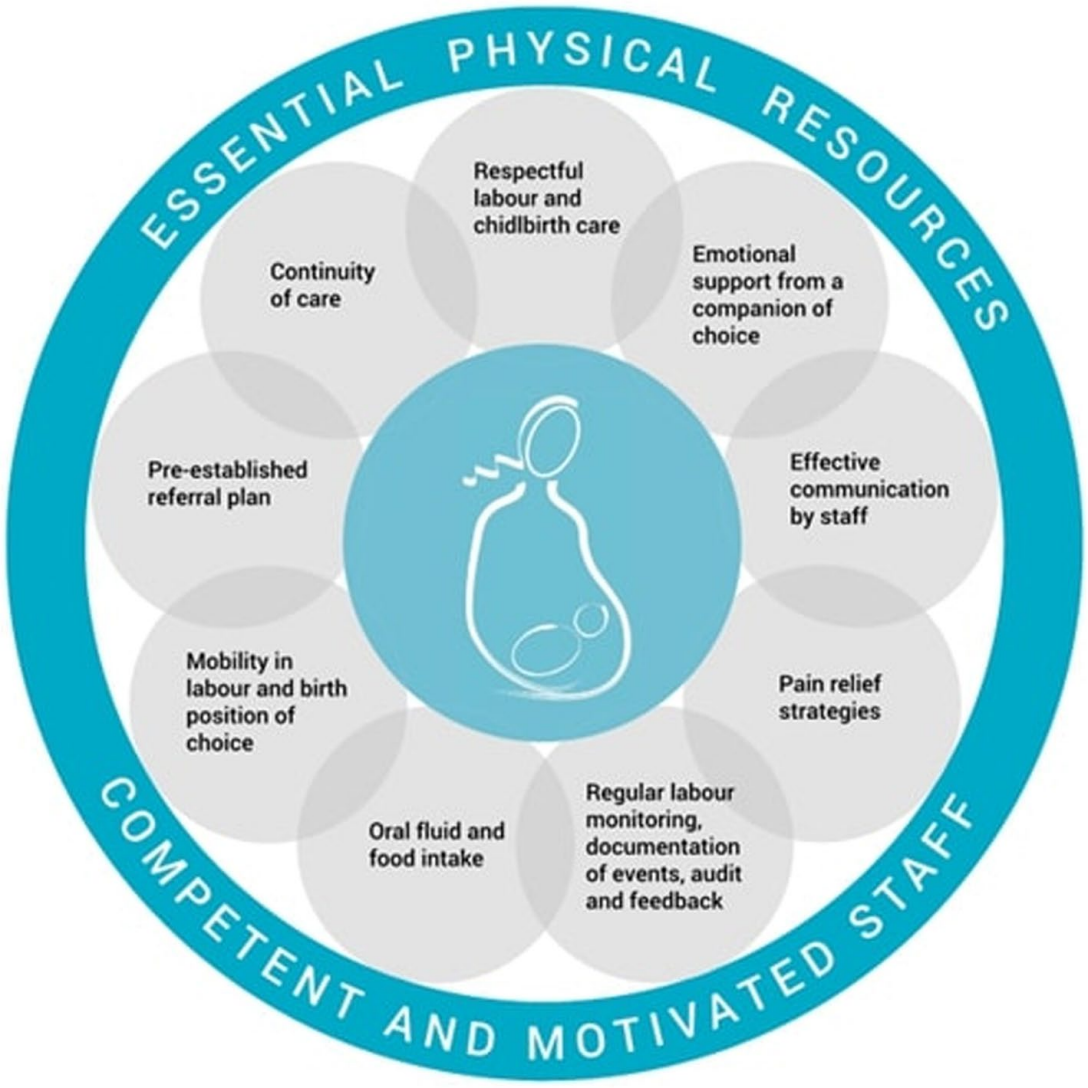
WHO intrapartum care model. Acknowledgements: WHO recommendations: Intrapartum care for a positive childbirth experience. Geneva: World Health Organization; page 169, Fig. 4.1, 2018. Available from: https://www.ncbi.nlm.nih.gov/books/NBK513809/. License: “CC BY-ND 2.0; https://creativecommons.org/licenses/by-nc-sa/3.0/igo/deed.en

As underlined by clinical practice and the before-mentioned recommendations, whatever the pregnant socio-economic status, race or other ethnic factors, care providers must guarantee collaboration between staff members, correct communication and support for the woman in addition to the competence and correct choice of the analgesic technique (see Fig. 1).

## COVID-19 pregnant

Although COVID19 infection can cause neurological localization and neuropathy, it is not a contraindication to NA that remains the safest and most effective analgesic approach in labor in this specific population. It avoids massive exposure to droplets exhaled by the patient during ventilation, intubation, and extubating maneuvers. However, it is desirable to have an experienced anesthesiologist to conduct it for both procedural and postpartum management due to the need for dedicated and protected environments and personal protective equipment (PPE) [46, 53].

## Expert opinion

Pain management during childbirth is a complex issue influenced by various social and cultural factors, making it impossible to address with medical knowledge alone. In recent years, there has been a shift in birth care programs aimed at overcoming the traditional, culturally shared view that associates childbirth with inhuman suffering. There is now a growing recognition of labor and the birthing process as complex and crucial experiences. Women have the right to participate in these events in a peaceful and constructive manner, having the ability to manage the circumstances and especially the pain they experience. In 2018, the WHO released a report titled “Intrapartum Care for a Positive Childbirth Experience,” which emphasizes that pharmacological strategies for managing pain during labor are an essential right of the parturients. This position was reaffirmed in 2020 amid updates related to the COVID-19 pandemic, asserting that all women have the right to a positive childbirth experience, regardless of whether they currently have a COVID-19 infection [1, 61]. The benefits established for mothers following NA include excellent pain control, reduced oxygen consumption, decreased hyperventilation, management of metabolic acidosis, lower levels of catecholamines and stress hormones, improved placental circulation, reduced anxiety, and, importantly, more relaxed and cooperative mothers. Furthermore, newborns also benefit from EA in several ways that reflect the advantages experienced by mothers. Specifically, these benefits include reduced metabolic acidosis, improved placental circulation due to vasodilation, decreased oxygen consumption, and enhanced oxygenation. Despite the benefits of labor analgesia, there is an ongoing debate about its impact on labor dynamics and the physiological completion of childbirth. The information available in scientific literature on this topic is often confusing and inconclusive. Much of the data comes from retrospective studies that include diverse populations and focus solely on anesthesiological variables, neglecting other healthcare disciplines involved in the childbirth process. Additionally, the partogram used during labor analgesia should be specifically designed for this purpose, rather than simply adapting the classical partogram for medicalized delivery. In Italy, there is limited published epidemiological data on childbirth analgesia, and it appears that anesthesiologists are often placed in a subordinate role compared to gynecologic surgeons. For instance, during discussions regarding the distribution of birth points, anesthesiologists were not invited to the working meetings. Additionally, the national quality indicators used by the Italian National Health System do not include criteria related to anesthesiology. However, anesthesiological work is essential throughout the entire process of childbirth (prepartum, during delivery, and postpartum). Limiting the anesthesiologist’s role to only the neuroaxial procedure undermines the significance of a complex process that involves pain control and outcome improvement. Based on our dated and established experience, we confidently state that:

- Optimal analgesia during childbirth must be “Tailored” for both mother and fetus because there are two patients to consider.
- Childbirth analgesia should not start when the technique is performed; rather, it should begin as soon as the pregnant woman enters the birth pathway. The woman must be included in the timeline, as defined by established protocols and maternal–fetal indications.
- The mother’s request for pain relief is enough to initiate analgesia during delivery, as defined in 2000 by the American Society of Anesthesiologists and ACOG; the timing should not be determined solely on cervical dilation [62].
- Active labor is sufficient to justify the administration of analgesia.
- Childbirth analgesia should be fully integrated into the care process, with an anesthesiologist present in the delivery room not only for emergencies, but for ongoing support.
- Avoid giving single-shot spinal analgesia late in the first stage of labor, as a catheter that allows for continuous analgesia or conversion to anesthesia in case of complications may be crucial for effective pain management.
- Properly managed top-up analgesia does not prolong the first active phase of labor; in fact, it can shorten it without negatively impacting the second stage of labor.
- Having a solid understanding of pharmacology in pregnancy enables skilled professionals to provide “tailored” analgesia. This approach aims to create a true dissociation from pain, selectively inhibiting nociception while still allowing for the transmission of information related to proprioception, epicritic, and protopathic sensitivity. This ensures that the parturient can participate fully and pain-free in the birthing process.
- NA techniques result in better pain control than other methods, as well as greater maternal satisfaction and better neonatal outcomes, and there is no evidence of increased incidence of cesarean section or vaginal operative deliveries.

In conclusion, NA becomes the “gold standard” only when it is tailored to a properly informed parturient, and a delivery room team with shared multidisciplinary training approaches the legitimate maternal request for assistance in a professional and empathic manner with clear, effective communication free of bias. Future studies should be developed through collaborative efforts among all professionals involved in the care of parturient. This cooperation will allow us to generate data aimed at enhancing our care.

## Conclusions

This review has the purpose of summarizing the main recent knowledge on labor analgesia. All described techniques are effective and safe and differ from each other regarding complications and adverse events. Despite this, as appears from recent recommendations, it is necessary to associate specific non-technical skills with technical skills and consider the operators’ expertise.

More trials and new strategies are needed to choose the better analgesic technique and to improve maternal–fetal safety. Moreover, future studies could clarify and implement the application of the new technologies in the obstetric care. In our opinion, NA is the best approach when it is tailored to the properly informed parturient, and managed by a trained multidisciplinary team.

## Data Availability

No datasets were generated or analysed during the current study.

## Abbreviations

NA: Neuraxial analgesia
WHO: World Health Organization
EA: Epidural analgesia
CSE: Combined spinal-epidural
DPE: Dural puncture epidural
CSA: Continuous spinal anesthesia
N_2_O: Nitrous oxide
TENS: Transcutaneous electric nerve stimulation
RMC: Respectful maternity care
RCT: Randomized controlled trials
LA: Local anesthetic
NICU: Neonatal intensive care unit
IDP: Inadvertent dural puncture
HELLP syndrome: Hemolysis, elevated liver enzymes, and low platelets
PDPH: Post-dural puncture headache
ACOG: American College of Obstetricians and Gynecologists
LAST: Local anesthetics systemic toxicity
PPE: Personal protective equipment
NSAIDs: Non-steroidal anti-inflammatory drugs
VR: Virtual reality
AI: Artificial intelligence
ERMF: Epidural-related maternal fever
PIEB: Programmed-intermittent epidural bolus
CEI: Continuous epidural infusion
PCEA: Patient-controlled epidural analgesia
